# The Pathophysiology of Osteoporosis after Spinal Cord Injury

**DOI:** 10.3390/ijms22063057

**Published:** 2021-03-17

**Authors:** Ramsha Shams, Kelsey P. Drasites, Vandana Zaman, Denise Matzelle, Donald C. Shields, Dena P. Garner, Christopher J. Sole, Azizul Haque, Narendra L. Banik

**Affiliations:** 1 Department of Neurosurgery, Medical University of South Carolina, 96 Jonathan Lucas St., Charleston, SC 29425, USA; shams@musc.edu (R.S.); drasites@musc.edu (K.P.D.); zamanv@musc.edu (V.Z.); matzeldd@musc.edu (D.M.); donshields@sbcglobal.net (D.C.S.); 2 Department of Microbiology and Immunology, Medical University of South Carolina, 173 Ashley Avenue, Charleston, SC 29425, USA; 3 Department of Health and Human Performance, The Citadel, 171 Moultrie St., Charleston, SC 29409, USA; garnerd1@citadel.edu (D.P.G.); csole@citadel.edu (C.J.S.); 4 Ralph H. Johnson Veterans Administration Medical Center, 109 Bee St., Charleston, SC 29401, USA

**Keywords:** bone loss, RANKL, Wnt, Osteoprotegerin (OPG), Estrogen (E2), neurodegeneration

## Abstract

Spinal cord injury (SCI) affects approximately 300,000 people in the United States. Most individuals who sustain severe SCI also develop subsequent osteoporosis. However, beyond immobilization-related lack of long bone loading, multiple mechanisms of SCI-related bone density loss are incompletely understood. Recent findings suggest neuronal impairment and disability may lead to an upregulation of receptor activator of nuclear factor-κB ligand (RANKL), which promotes bone resorption. Disruption of Wnt signaling and dysregulation of RANKL may also contribute to the pathogenesis of SCI-related osteoporosis. Estrogenic effects may protect bones from resorption by decreasing the upregulation of RANKL. This review will discuss the current proposed physiological and cellular mechanisms explaining osteoporosis associated with SCI. In addition, we will discuss emerging pharmacological and physiological treatment strategies, including the promising effects of estrogen on cellular protection.

## 1. Introduction

In the United States, 17,730 new cases of spinal cord injury (SCI) are reported annually; 78% of these are males. Vehicular crashes account for the largest proportion of SCI cases (about 39.3%) in 2019. SCI cases have also been significantly attributed to falls, acts of violence, such as gunshot wounds, and athletic injuries. Less than 1% of individuals with severe SCI experience full neurological recovery. Moreover, SCI can have chronic implications with constant management and lifestyle readjustments [1,2]. There is currently no effective FDA-approved treatment for SCI; therapies are largely directed toward management of motor impairments and associated symptoms. The lifetime indirect costs of SCI in the United States may range from $ 0.3 million to $ 1.3 million, depending on the age of the SCI individuals [3]. 

With respect to clinical staging, the primary stage of SCI includes mechanical trauma to the spinal cord [4,5,6]. Modes of primary injury include compression, laceration, and contusion with resulting vascular injury, direct mechanical injury to neurons, and subsequent axonal degeneration. This cellular damage at the lesion site is largely irreversible. The secondary stage of SCI is characterized by multiple cascading events within hours of the primary stage. The resulting tissue degeneration, neuroinflammation, and cell death can increase the lesion size and limit restorative processes without effective intervention [4,7]. In particular, secondary stage injury associated inflammation influences the reduction of blood flow to the spinal cord, activation of chemokines and cytokines, upregulation of Calpain proteases, degeneration of the blood brain barrier, and accumulation of free radicals. Therefore, secondary stage injury drives chronic morbidity (e.g., paralysis) through complicated mechanisms leading to axonal degeneration, demyelination, ischemia, and necrosis of spinal cord tissue [4,6]. 

Individuals with SCI are at an increased risk of developing complications associated with paralysis-related inactivity; they often manifest rapid and severe bone density loss [6,8]. This bone loss occurs in two phases. The first phase is characterized by rapid bone reabsorption, which plateaus 18–24 months post-injury. The second chronic phase is characterized by gradual bone loss and inhibited bone formation [9,10]. As a result, about 40% of individuals with chronic SCI experience fractures; thus, twice the likelihood of fracture formation compared to individuals without SCI [9]. 

SCI affects the sympathetic nervous system through compromise of neuronal afferent connections [11]. Therefore, intravenous shunts develop throughout the bone, and venous/capillary blood flow is hindered. Consequently, decreased gas exchange and nutrient delivery promotes local hyperpressure and increased stimulation of osteoclasts. Significant bone resorption and demineralization also occurs [10,11]. This mechanism suggests a link between neural and physiological disorders in the pathogenesis of bone loss [11,12]. The molecular and cellular mechanisms driving the pathogenesis of osteoporosis in SCI are not well characterized, but Wnt signaling and receptor activator of nuclear factor-κB (RANK)/receptor activator of nuclear factor-κB ligand (RANKL)/osteoprotegerin (OPG) signaling have been implicated [12,13,14].

Sympathetic tone is also variably altered depending on severity of injury and the level of SCI in the cervical, thoracic, or lumbar spine. Bilateral sympathetic trunks (chains) receive preganglionic inputs from T1–L3 cord levels. Injuries to cervical and upper thoracic levels (T1–6) can cause neurogenic shock with corresponding decreased vascular tone, hypotension, bradycardia, temperature dysregulation, etc. Although incompletely understood, cervical and upper thoracic SCI may cause more significant long-term sympathetic/parasympathetic imbalances compared to lower thoracic and lumbar injuries. Norepinephrine interacts with β2-adrenergic receptors in bone to affect osteogenesis and bone resorption in a common pathway with parathyroid hormone [15]. Rodent models demonstrate alveolar bone loss with decreased sympathetic input [16]. Moreover, sympathetic inhibition and β2-adrenergic receptor blockade decrease the number of resident bone marrow effector memory T lymphocytes, which contribute to hypertension [17]. Thus, SCI level and corresponding autonomic dysfunction contribute to skeletal homeostasis.

Individualized treatments are complicated by current limited understanding of the mechanisms associated with SCI osteoporosis. There are no clinical guidelines for prevention and treatment of osteoporosis after SCI [13,18]. However, approaches incorporating physical activity have demonstrated promise in increasing bone mass in osteoporosis patients [19]. It is suggested that modulating the Wnt pathway via mechanical loading contributes to the potential therapeutic properties of physical activity [9,13,20,21]. For individuals suffering from paralysis, electrical stimulation therapies have been developed [22,23]. However, more feasible clinical approaches have been investigated; several groups have explored the efficacy of several pharmacological inhibitors in treating osteoporosis in individuals with SCI. These options include cathepsin K inhibitors, sclerostin (SOST) inhibitors, and recombinant parathyroid therapy. However, these approaches can be ineffective for bone growth while placing the individual at risk for potential off-target effects [24]. Recently, treatments targeting the RANK pathway, such as Denosumab and estrogen hormone replacement therapy, have demonstrated promising outcomes for people suffering from SCI [13].

## 2. Mechanisms Regulating Bone Remodeling

Bone regeneration is influenced by a relationship between osteoblasts and osteoclasts. Osteoblasts (derived from mesenchymal cells) generate new bone. Osteoclasts are derived from monocyte macrophage precursors and are responsible for bone resorption. Osteoblasts secrete macrophage-colony stimulating factor (M-CSF) and RANKL [11,25,26]. RANKL binds to the RANK receptor on osteoclast precursors, stimulating the formation of multinucleated cells [11,26]. In a complicated signaling pathway, transcription factors nuclear factor-κB (NF-kB), c-Fos, and nuclear factor of activated T cells C1 (NFATc1) are activated-ultimately stimulating osteoclast differentiation [27,28,29,30]. When activated, these differentiated osteoclasts attach to the bony surfaces. They secrete hydrochloric acid to dissolve bone mineral and cathepsin K to dissolve bone matrix. Thus, bone structural units are formed when discrete sections of bones are dissolved by cooperating groups of osteoclasts [11]. After resorption, the reversal phase occurs, in which osteogenic molecules are recruited to foster a microenvironment promoting bone formation, thereby assisting the coupling of bone formation to bone resorption [31]. Upon the recruitment of osteoblast progenitors [32,33], the process of bone formation occurs. A thin layer of mononuclear cells lines the bone surface, forming a reversal line, and bone collagen matrix is layered over the reversal line by osteoblasts. While some calcify, other osteoblasts are integrated into the matrix, forming tight connections with the bone surface and osteocytes through cytoplasmic interactions [11]. Therefore, bone resorption and formation proceed as coupled processes, an essential co-interaction that maintains bone integrity [9,11]. 

In individuals with SCI, when bone formation/resorption becomes uncoupled, the result is lower bone mass and decreased bone integrity, osteoporosis [11]. In SCI, the mechanisms for osteoporosis onset are largely distinct from osteoporosis arising from disease, malnutrition, or pharmacological side effects [34]. Demulder et al. suggested that SCI may promote secretion of compounds that stimulate osteoclastogenesis. In a project studying individuals with SCI (paraplegia), an increased number of osteoclast-like cells in the iliac bone marrow culture compared with sternal bone marrow culture (as well as higher amounts of interleukin 6 (IL-6) in the iliac conditioned media) were reported [35,36]. IL-6 has been correlated to the stimulation of osteoclast-like cell formation through the secretion of interleukin 1β (IL-1β) by local monocyte-macrophages in the marrow microenvironment [37]. This suggests motor impairments, such as paraplegia, may promote the formation of osteoclast-like cells and their progenitors [35]. 

Mechanical loading plays a crucial role in bone remodeling. Bones can adapt to variations in weight-bearing and mechanical loading. Likewise osteocytes can detect mechanical strain and regulate adaptive bone remodeling through biochemical signals [38]. Mechanical unloading, due to limb paralysis for example, fails to stimulate osteocyte modulation of osteoblast/osteoclast activity. Furthermore, the Wnt signaling pathway orchestrates bone remodeling at the molecular level in response to mechanical loading. Dysregulation of the Wnt and RANKL/RANK/OPG pathways are implicated in the development of osteoporosis in individuals with SCI [39,40,41].

The risk of developing osteoporosis is further exacerbated by deficiencies of Vitamin D (25(OH)D); this is more prevalent in populations with chronic SCI than the general population [42,43]. Medication, institutionalization, and lifestyle changes leading to decreased sun exposure are possible risk factors. Moreover, vitamin D promotes reabsorption of calcium in the gut. When vitamin D levels are insufficient, individuals with SCI may develop iatrogenic hypocalcemia if antiresorptive therapies are used [42]. 

## 3. Cellular and Molecular Pathways Resulting in Osteoporosis after SCI

The cellular and molecular pathways resulting in osteoporosis development after SCI have not been fully elucidated [44]. However, recent studies have expanded the understanding of how mesenchymal stem cells (MSCs) influence bone development at the cellular and molecular levels [44]. MSCs are pluripotent cells and differentiate into a variety of cell types: myoblasts, chondrocytes, osteoblasts, and adipocytes. The maturation into these cell types is dependent on growth factor and signaling via multiple pathways (Wnt, fibroblast growth factor (FGF), bone morphogenetic protein (BMP)/ transforming growth factor (TGF), etc.). Cell fate decisions for progenitor cells are also not explicitly defined; however, downstream transcription factors and the epigenetic landscape may influence the decision to commit to the respective tissue type. 

The fibroblast growth factor (FGF) signaling cascade is influential in MSC cell fate decisions [45]. Transduction of FGF signaling occurs through the activation of the extracellular signal-regulated kinase 1/2 (ERK1/2), p38 mitogen-activated protein kinase (p38 MAPK), protein kinase C (PKC), stress-activated protein kinase/c-Jun NH(2)-terminal kinase (SAPK/JNK), and phosphoinositide 3-kinase (PI3K) pathways [46]. FGF signaling may work in coordination with other signaling cascades. For example, studies have correlated members of the transforming growth factor-β (TGF-β) superfamily to enhanced osteogenesis [47]. Bone morphogenetic proteins (BMPs) are structurally related to TGF-β superfamily, and they may also facilitate the differentiation of MSCs into osteoprogenitor cells [48].

As previously discussed, downstream transcription factors may influence cell fate decisions. For example, peroxisome proliferator-activated receptor-γ (PPARγ) and CCAAT-enhancer-binding proteins (C/EBPs) have been elucidated as key players in adipogenic differentiation of MSCs [49]. For osteogenic differentiation of MSCs, runt-related transcription factor 2 (Runx2), and Osterix have been identified as crucial transcription factors [50,51]. Particularly, upregulation of *Runx2* promotes the differentiation of MSCs into immature osteoblasts [52]. The activity of Runx2 is facilitated by increased glucose uptake via Glut1 activity and is mediated by the upregulation of members of the FGF family [45,53]. Immature osteoblasts can be differentiated into mature osteoblast in the presence of Osterix, and they express hallmark osteoblast markers such as type 1 collagen, osteocalcin, osteonectin, and osteopontin [51].

In SCI, the cell fate decisions of MSCs are interesting, particularly in regards to the commitment to adipocytes versus osteoblasts. Interestingly, individuals with SCI often demonstrate a relatively higher body fat composition compared to subjects without SCI. Adipocytes are typically considered osteoprotective; however, individuals with SCI experience increased bone fragility and are more likely to develop bone fractures [13,41]. Studies have suggested that, after SCI, there is increased differentiation of MSCs into adipocytes rather than osteoblasts, which may be induced by overexpression of peroxisome proliferator-activated receptor-γ (PPARγ) [44,54,55,56]. Studies also demonstrate PPARγ expression may increase bone resorption [44]. Additionally, when osteoblasts demonstrate PPARγ expression, the mature osteoblast phenotype is suppressed, and osteoblasts express genes such as lipoprotein lipase (LPL), fatty acid binding protein 4 (FABP4/aP2), and fatty acid synthase (FAS) [44]. Therefore, adipocytogenesis is promoted rather than osteoblastogenesis. Thus, individuals with SCI may not benefit from the osteoprotective function of adipocytes, and continued bone loss is observed [44].

### 3.1. Wnt Pathway

The Wnt signaling pathway is a highly conserved signaling pathway with several roles in biological processes, such as in animal development and cell fate decisions. The Wnt pathway is composed of 19 secreted glycoproteins [57]. Two proteins, Porcupine (Porc) and Wntless (Wls), are essential to the secretion of Wnt proteins. Porc is an acetyltransferase located on the endoplasmic reticulum and is responsible for mediating lipidation of the Wnt protein following synthesis [58,59]. Wls, a transmembrane, is responsible for Wnt secretion [58,60]. Secreted Wnt proteins may activate either the β-catenin-dependent canonical Wnt pathway or the β-catenin-independent non-canonical Wnt pathway.

Wnt is crucial in the activity of β-catenin. When Wnt stimulation is suppressed, a complex of Axin, glycogen synthase kinase-3 β (GSK-3β), and adenomatous polyposis coli (APC) phosphorylates cytoplasmic β-catenin. Phosphorylated β-catenin is ubiquitinated, and proteasomal system rapidly degrades the ubiquitinated β-catenin. However, upon secretion of Wnt, the Wnt ligands bind to the 7-transmembrane domain-spanning Frizzled receptor and low-density lipoprotein receptor-related proteins (LRP-5 and LRP-6), the three of which form a co-receptor complex. Binding of Wnt ligands prevents phosphorylation of β-catenin via suppression of GSK-3β activity. Thus, binding of Wnt facilitates the stabilization of β-catenin, inducing β-catenin in the cytoplasm. Subsequently, unphosphorylated β-catenin translocates into the nucleus and regulates gene expression with the T cell factor (TCF)/lymphocyte enhancer factor 1 (LEF1) and cAMP response element-binding protein (CBP) complex [61,62].

### 3.2. Wnt Pathway in Bone Homeostasis

**Figure 1:**
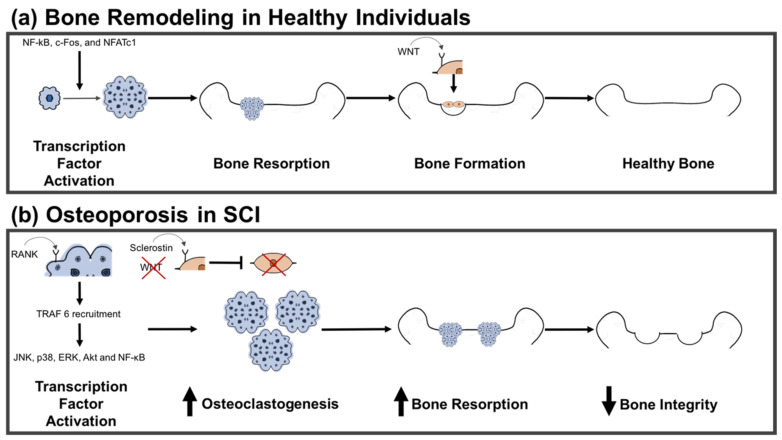
Typical bone remodeling vs. bone remodeling in individuals with spinal cord injury (SCI). (**a**) In individuals without SCI, bone remodeling is typically a balanced process. Transcription factors nuclear factor-κB (NF-kB), c-Fos, and nuclear factor of activated T-cells, cytoplasmic 1 (NFATc1) secreted by osteoblasts are activated, stimulating the maturation of mononucleated osteoclast progenitors. Mature osteoclasts secrete cathepsin K and hydrochloric acid (HCl) to dissolve the bone matrix, with resulting calcium resorption. Moreover, Wnt protein can bind to receptors on osteoblasts. Upon activation, osteoblasts form a layer across the surface of the dissolved bone for calcification. When these processes are coupled, bone integrity is preserved. (**b**) In individuals with SCI, bone remodeling is no longer balanced, resulting in osteoporosis. Receptor activator of nuclear factor-κB (RANK) ligand binds to RANK receptors on osteoclasts, inducing TNF receptor-associated factor 6 (TRAF-6) recruitment and activation of transcription factors c-Jun N-terminal kinase (JNK), p38, extracellular signal-regulated kinase (ERK), alpha serine-threonine protein kinase (Akt) and NF-κB. These factors participate in various cellular signaling pathways to induce osteoclastogenesis. Furthermore, Wnt protein may be unable to bind due to the presence of Wnt-receptor competitive inhibitors (e.g., sclerostin (SOST)), preventing osteoblast activation. Therefore, increased bone resorption occurs without osteoblast-mediated bone construction. Bone integrity is compromised with resulting osteoporosis.

In particular, studies have deduced the implications of the Wnt pathway in bone homeostasis related to mechanical loading. Interestingly, osteoblasts have Frizzled receptor, LRP-5, and LRP-6 co-receptor complex. Wnt binds to the receptor in osteoblasts, which in turn promotes the translocation of cytoplasmic β-catenin into the nucleus of the osteoblast (Figure 1). Consequently, translocation activates transcription of genes enabling osteoblast differentiation and activity with resulting bone formation [13,44]. 

Studies have implicated Wnt ligands with specific functions in bone homeostasis. For example, Wnt3a may have the role of activating TAZ by PP1A-mediated dephosphorylation, stimulating osteogenic differentiation [63]. Similarly, Wnt10b overexpression may increase trabecular bone density by enhancing osteoblastogenesis [64]. Wnt10b deficiency may decrease bone density, while promoting adipogenic differentiation of MSCs [65,66]. Additionally, through the canonical Wnt pathway, *Wnt6, Wnt10a, and Wnt10b* facilitate the differentiation of MSCs to osteoblasts while suppressing adipogenic differentiation of MSCs [62]. 

Several in vivo studies have been performed to elucidate the roles of Wnt in bone homeostasis. Luther et al. investigated Wnt1 knockout in osteoblasts in mice and demonstrated a decrease in bone volume. However, in osteoblast-targeted inducible Wnt1 transgenic mice, Wnt1 induction increased trabecular and cortical bone mass rapidly, while stimulating bone formation [67]. Joeng et al., developed osteoblast-specific and osteocyte-specific Wnt1 loss- and gain-of-function in mouse models and demonstrated an increase in spontaneous fractures, a decrease bone mass in both male and female, and a decrease in bone formation rate, suggesting the importance of Wnt signaling in regulating bone loss [68]. Yu et al. studied transgenic expression of Wnt4 in osteoblasts and suggested that bone volume and bone formation are increased [69]. Maeda et al. showed impaired osteoclastogenesis following Wnt5a knockout in mice, leading to increased bone mass and a reduction in bone resorption, via the non-canonical Wnt pathway [70]. Using *Wnt7b* transgenic mice, Chen et al. also deduced the role of Wnt7b in promoting bone formation through activation of mTORC1 in osteoblasts and subsequently increasing bone mass in mice [71]. As previously discussed, it has been proposed by the Bennet et al. lab that Wnt10b plays a crucial role in stimulating osteoblastogenesis, leading to significantly increased bone mass density, bone volume fraction, and trabecular number in transgenic mice [64]. Wnt10b may stimulate osteoblastogenesis by suppressing PPARγ expression [72]. Movérare-Skrtic et al. demonstrated that knockdown of *Wnt16* in mouse osteoblasts increases fracture susceptibility by decreasing cortical thickness and enhancing bone resorption [73]. 

Several antagonists for the Wnt pathway have been identified [9,74,75,76]. The Dickkopf (DKK) protein family, ubiquitously expressed in vivo, has been identified as an inhibitor of Wnt signaling. DKK1 is a competitive inhibitor of Wnt, binding to BP1 and BP3 domains on the LRP5/6 receptor. DKK1 binding subsequently induces internalization of LRP5/6 receptor, inhibiting the canonical Wnt pathway [77,78]. In DKK1 knockout mice, bone formation and osteoblast number are drastically increased, contributing to elevated trabecular and cortical bone masses in mice [79]. In addition, SOST inhibits the canonical Wnt pathway by competitively binding to the BP1 domain on the LRP5/6 receptor [62]. Therefore, SOST binds onto Wnt, and results in the formation of Wnt-LRP5-Frizzled complex [20,80,81]. SOST is of particular interest, because it is primarily secreted by osteocytes. SOST is associated with catabolic and anti-anabolic effects in bone. In vivo studies have demonstrated that, when *SOST* is suppressed, mice will exhibit increased bone mass and decreased bone fragility due to enhance osteogenesis [82]. Studies have also correlated *SOST* to the upregulation of RANKL and downregulation of osteoprotegerin (OPG), increasing osteoclast activity and subsequent bone resorption. Additionally, mechanical unloading, such as in SCI, reduces Wnt signaling in osteoblasts through the upregulation of *SOST* [13,20]. SOST is typically suppressed by parathyroid hormone (PTH). However, in acute and chronic SCI, dysregulation of PTH stimulates *SOST* expression. 

## 4. The RANK/RANKL/OPG System Is Influential in Bone Health

For decades, the RANKL/RANK/OPG system has been understood as a crucial modulator for bone resorption [41,83]. The successful binding of RANKL to its receptor, RANK, promotes the differentiation, activation, and survival of osteoclasts [41,83]. 

### 4.1. Osteoprotegerin (OPG) 

OPG is a member of the tumor necrosis factor (TNF) superfamily [84]. This peptide is composed of 401 amino acids, and when cleaved, the resulting mature form contains 380 amino acids [84]. The structure of OPG does not have cytoplasmic and transmembrane domains. Many tissues express OPG, including lung, muscle, and bones [84]. However, for many of these tissue types, the role of OPG has yet to be fully characterized [85,86]. OPG is known to act as a competitor for RANKL when expressed in its soluble form [87]. Studies have suggested OPG has an osteoprotective role since overexpression of OPG demonstrates an osteopetrosis phenotype, while OPG deficiency promoted osteoporosis development [88,89]. 

### 4.2. RANK and RANKL

Human RANK is a receptor comprised of 616 amino acids and a 28-amino acid signaling peptide [90]. RANK has a 184-amino acid N-terminal extracellular domain, a 383-amino acid C-terminal cytoplasmic domain, and a 21-amino acid transmembrane domain [90]. RANK is expressed by macrophage and monocyte-like cells, such as T cells, B cells, fibroblasts, dendritic cells, and preosteoblastic cells [90]. Additionally, RANK is expressed on the cell surface of osteoclasts and their progenitors [89]. In a study using RANK knockdown mice, osteoclast expression was inhibited with osteopetrosis development [41,91]. 

RANKL is also a member of the TNF superfamily, and demonstrates high conservation amongst species [84,92,93]. In humans, the RANKL gene is located on chromosome 13q14, spanning approximately 36 kb of DNA [90]. Translated human RANKL is a peptide consisting of 317 amino acids, folding into a 45 kDa membrane-associated protein [94]. Soluble RANKL has a molecular weight of 31 kDa, and is expressed in several different tissue types including lymphoid tissue, mammary glands, and bone [94]. In bone, RANKL is expressed in osteoblast, osteoblast precursors, and stromal cells [95,96]. Recent studies have suggested that osteocytes express high levels of RANKL in order to activate osteoclastogenesis. Knockdown models of RANKL expression in osteocytes demonstrate inhibition of osteoclast activity, increased bone density, and severe osteopetrosis [41,91,93].

### 4.3. The Role of RANKL/RANK/OPG in Osteoclastogenesis

Factors such as PTH, vitamin D, and prostaglandin 2 stimulate osteoclastogenesis through the presentation of RANKL to osteoclastic progenitor cells by osteoblasts or osteocytes [97,98,99,100]. Soluble or membrane-bound RANKL binds to RANK embedded on the surface of these osteoclastic precursors, and this receptor-mediated interaction initiates the recruitment of several adaptor proteins, such as TNF receptor-associated factor 2 (TRAF2), TRAF5, and TRAF6 to the RANK membrane-proximal domain [101,102,103]. TRAF6 is essential to osteoclastogenesis, and studies have demonstrated that TRAF6 is the only member of the TRAF family whose deficiency exhibits an osteopetrosis phenotype [102,103]. Subsequently, TRAF6 recruitment initiates the activation of multiple molecules involved in signaling pathways, including JNK, p38, ERK, Akt and NF-κB [104,105]. The processes involved in the activation of NF-κB, p38-ERK, and JNK interact with NFATc1 in the cell nucleus, stimulating the transcription of genes crucial for osteoclastogenesis [106,107,108]. Mononucleated osteoclast progenitors fuse, and RANKL mediates reorganization of the cytoskeleton for osteocyte maturation and subsequent bone-resorbing activity (Figure 1) [109,110].

### 4.4. Dysregulation of RANK Ligand in SCI-Related Osteoporosis

As previously discussed, bone remodeling is reliant on the coupled actions of osteoblasts/osteoclasts, bone resorption and bone formation. Bone loss after SCI is induced by decreased bone formation and increased bone resorption, which results in compromised bone microarchitecture and structural integrity. Though poorly understood, studies suggest that SCI promotes RANK/RANKL overexpression with increased expression in osteoclasts. Studies have also demonstrated decreased OPG-positive cells, promoting RANK/RANKL signaling [111,112]. As previously discussed, PPARγ expression is increased in individuals with SCI, which is associated with the increased expression of RANKL [44].

### 4.5. RANKL/OPG Regulation Using Estrogen

In both male and female subjects, estrogen (E2) is a promising therapeutic agent demonstrated to reduce pro-inflammatory activities and enhance recovery in SCI [113,114]. Concerns, however, arise with the systemic administration of E2 and presumptive off-target effects, particularly in males [115]. However, it is demonstrated that E2 administered in lower doses nearly close to physiological levels is enough to facilitate recovery via the protective function of E2, while limiting the development of adverse side effects [116,117,118,119] irrespective to sex [120]. 

Studies have suggested E2 plays a significant role in regulating RANKL [121,122]. In osteoporosis studies, post-menopausal women (lower E2 expression) are used as a model, because they are susceptible to osteoporosis and bone atrophy related fractures [123,124]. Studies have demonstrated lower RANKL levels in the bone marrow of E2-treated post-menopausal women compared to those who were not given E2 treatment, suggesting a protective effect of estrogen [125]. 

**Figure 2:**
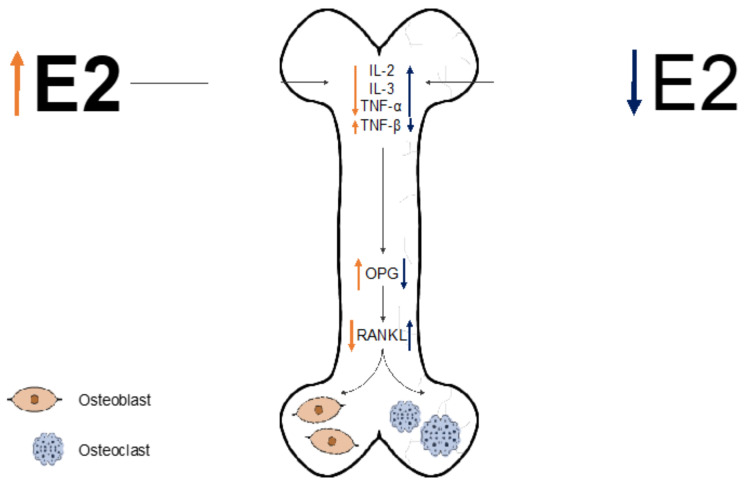
Protective effects of estrogen (E2) against osteoporosis. When there are low levels of E2 in the body, interleukin 2 (IL-2), interleukin 3 (IL-3), and tumor necrosis factor (TNF)-α are upregulated, while TNF-β is downregulated. The concerted effects of these increased levels of cytokines induce downregulation of competitive osteoprotegerin (OPG) and the upregulation of receptor activator of nuclear factor-κB ligand (RANKL). Increased RANKL contributes to osteoclast differentiation, subsequently increasing bone resorption. Inversely, when there are higher levels of E2 in the body, IL-2/IL-3/TNF-α are downregulated, and TNF-β is upregulated-initiating a cascade of increased OPG and decreased RANKL expression. Osteoblast differentiation occurs at an increased pace (preserving bone integrity), particularly in osteoporosis-prevalent conditions, such as SCI. Orange arrows indicate pathway governing osteoblastogenesis, while blue arrows indicate pathway governing osteoclastogenesis.

Due to the loss of E2, pro-inflammatory cytokines IL-1, IL-6, and TNF-α are upregulated, and TNF-β is downregulated [126,127,128,129]. The combined effect of these processes stimulates osteoclastic activity, resulting in abnormal bone loss (Figure 2) [126,127,128,129]. Inversely, in human osteoblasts, high levels of E2 increase OPG expression and decrease RANKL expression [121,130]. OPG inhibits RANKL function, therefore stimulating bone formation (Figure 2) [131]. Further, E2 is influential in the lifespan of osteoblasts and osteoclasts. E2 stimulates osteoblast production of TGF-β, which induces osteoclast apoptosis [132]. Similarly, osteoclast E2 receptor activation promotes a proapoptotic effect, shortening osteoclast life span and inhibiting bone-resorbing function [132,133]. E2 has been found to have beneficial effects upon inflammation, oxidative damage, angiogenesis, cell death, and functional improvement in animal models of SCI [134,135]. Thus, effective dosing of E2 may have potential therapeutic importance in SCI treatment paradigms.

## 5. Physical Exercise to Combat Osteoporosis

Multiple studies in people with SCI suggest physical activity is beneficial [19], particularly in bone mass outcomes [136,137]. Muscular training can enhance blood flow, which in turn, enhances bone vasculature. Femoral blood flow, for example, approximately doubles in response to exercise [138]. Additionally, blood flow to bones occurs in response to changes in metabolic activity, and SCI-associated osteoporosis can be positively affected by physical activity via enhanced bone metabolism and regeneration [21,139]. As previously discussed, the Wnt signaling cascade is perturbed due to limb unloading and muscular disuse-promoting osteoporosis [9,140,141]. 

Weight-bearing exercises may reverse the effects of Wnt dysregulation through mechanical loading [9,21]. Normally, the skeleton responds to increasing mechanical strain by increasing cortical bone tissue localized to the site of the mechanical strain [142]. However, since weight-bearing exercise is difficult to implement and may require a substantial daily time investment in many individuals with SCI [90,143], electrical stimulation (ES), and functional electrical stimulation (FES) have been suggested to be effective options by innervating muscles using directed electrical stimulation [22,23]. Studies have demonstrated reduced bone resorption, preservation of trabecular bone micro-architecture, and stimulation of new bone formation after these treatments [136,144,145,146]. However, more studies are required to establish ES and FES as feasible clinical options, and furthermore, the literature largely fails to deduce a significant improvement in patient bone restoration following physical activity as the primary therapeutic intervention, enhancing the necessity of pharmacological agents in the treatment of SCI-related osteoporosis.

## 6. Pharmacological Strategies to Treat Osteoporosis

Currently, anti-resorptive medications (e.g., bisphosphonates) are prescribed to treat osteoporosis in individuals with SCI. Due to the inhibition of osteoclast recruitment leading to decreased bone turnover, bisphosphonates are effective in slowing bone loss in acute and chronic cases of SCI [142,146,147]. Clinical studies also suggest they may reduce fracture rate in participants with osteoporosis. However, bisphosphonates have been demonstrated to not be effective for increasing bone density [142,146,148]. Further, fracture reduction may not be sustained once treatment is stopped [147,148]. In bisphosphonate administration studies involving individuals with SCI, participants often report flu-like symptoms, such as body temperature elevation, myalgia, respiratory congestion, and fatigue, as well as urinary tract infection and constipation [149,150,151,152].

Cathepsin K is thought to be responsible for osteoclast-mediated bone loss, and corresponding inhibitors have been investigated as a treatment option for osteoporosis in post-menopausal women [153]. Cathepsin K inhibitors target bone resorption, while enabling bone formation to occur through osteoclast stimulation [153,154]. However, phase III clinical trials for the cathepsin K inhibitor, Odanacatib, have been halted due to increased cardiovascular events, such as strokes [155,156]. These results suggest that cathepsin K inhibitors may have negative off-target effects and may not be valuable for clinical use.

SOST is a promising therapeutic target. As previously discussed, SOST impedes Wnt signaling, therefore promoting the downregulation of osteoblastogenesis [157]. Additionally, the corresponding *SOST* gene is expressed exclusively by skeletal tissue, making it an attractive clinical target. Targeting the *SOST* gene and subsequently SOST protein would mitigate off-target effects while directly addressing skeletal health. Romosozumab is an anti-SOST monoclonal antibody that inhibits SOST, resulting in increased osteoblast proliferation [157,158]. Clinical trials with this agent have demonstrated maintained or increased bone mineral density, the stimulation of bone formation, and the inhibition of bone resorption [158,159].

PTH is an additional therapeutic target for its influence in osteoblastogenesis. Administration of a recombinant human PTH, Teriparatide, [160,161] have demonstrated a persistent reduction in fracture incidence, back pain, as well as improved bone architecture [162,163,164]. It is one of the few therapeutic agents available for osteoporosis that may increase bone mass and enhance bone architecture. However, the use of Teriparatide is limited to 24 months; repeated administration may lead to decreasing bone mass and the potential of detrimental side effects [163,165].

Some therapies target the RANKL/RANK/OPG pathway. Denosumab, a monoclonal antibody against human RANKL, inhibits binding and activation of RANK, inhibiting RANK-mediated osteoclastogenesis [125,166]. Although effective in limiting bone loss, competitive RANKL inhibition may have off target effects on immune function [167]. Further, as previously discussed, E2 inhibits bone loss, and hormone replacement therapy (HRT) of E2 or E2/progesterone combinations have successfully demonstrated osteoclast inhibition with bone mass restoration [168,169,170]. E2 HRT is however associated with an increased risk of breast cancer development and heart disease in women [171]. Nevertheless, recent studies suggest localized E2 administration, using a small yet effective dose, can mitigate the risk of cancer while also limiting the destructive pathways leading to osteoporosis [116,134].

## 7. Conclusions

The need for therapeutic modalities for individuals suffering from SCI continues, yet the mechanisms of osteoporosis in SCI have not been fully elucidated. This review attempts to summarize recently proposed physiological and cellular mechanisms mediating osteoporosis in respect to SCI. The Wnt pathway is crucial for its role in the maintenance of bone, and dysregulation of Wnt may promote the osteoporosis phenotype. Inhibitors targeting *SOST*, an inhibitor of the Wnt pathway, has demonstrated promise for increasing osteoblast proliferation. Studies have also explored physical activity as a potential strategy, and it has been demonstrated that load-bearing exercise and muscular innervation may alleviate the negative effects of Wnt dysregulation. However, these strategies are clinically infeasible for individuals with SCI. Additionally, RANKL/RANK/OPG system is highly influential in bone resorption, and in SCI, neuronal impairment and disability may lead to an upregulation of RANKL, a potential contributor to osteoporosis. Pharmacological treatment strategies targeting the RANKL/RANK/OPG system, such as Denosumab, inhibit binding and activation of RANK and mediate osteoclastogenesis. However, these agents are not effective and have off-target effects. Preclinical studies have suggested the success of small, localized doses of E2 on cellular protection in bone tissue, regulating RANKL expression. Future studies are necessary to explore the promising anti-inflammatory effects of E2 for overcoming bone loss and as a potential clinical candidate especially for treatment of individuals with chronic SCI.

## Data Availability

No new data were created or analyzed in this study. Data sharing is not applicable to this article.
